# Pathogen non-planktonic phases within the urinary tract impact early infection and resistance evolution

**DOI:** 10.1093/ismejo/wrae191

**Published:** 2024-09-26

**Authors:** Michael Raatz, Amanda de Azevedo-Lopes, Karolina Drabik, Arne Traulsen, Bartlomiej Waclaw

**Affiliations:** Department of Theoretical Biology, Max Planck Institute for Evolutionary Biology, 24306 Plön, Germany; Department of Theoretical Biology, Max Planck Institute for Evolutionary Biology, 24306 Plön, Germany; Dioscuri Centre for Physics and Chemistry of Bacteria, Institute of Physical Chemistry (IChF), Polish Academy of Sciences, 01-224 Warsaw, Poland; Department of Theoretical Biology, Max Planck Institute for Evolutionary Biology, 24306 Plön, Germany; Dioscuri Centre for Physics and Chemistry of Bacteria, Institute of Physical Chemistry (IChF), Polish Academy of Sciences, 01-224 Warsaw, Poland; School of Physics and Astronomy, University of Edinburgh, EH9 3FD Edinburgh, United Kingdom

**Keywords:** resistance evolution, evolution-informed medicine, antibiotic therapy, mathematical modeling

## Abstract

Treatment of urinary tract infections and the prevention of their recurrence is a pressing global health problem. In a urinary infection, pathogenic bacteria not only reside in the bladder lumen but also attach to and invade the bladder tissue. Planktonic, attached, and intracellular bacteria face different selection pressures from physiological processes such as micturition, immune response, and antibiotic treatment. Here, we use a mathematical model of the initial phase of infection to unravel the effects of these different selective pressures on the ecological and evolutionary dynamics of urinary infections. We explicitly model planktonic bacteria in the bladder lumen, bacteria attached to the bladder wall, and bacteria that have invaded the epithelial cells of the bladder. We find that the presence of non-planktonic bacteria substantially increases the risk of infection establishment and affects evolutionary trajectories leading to resistance during antibiotic treatment. We also show that competitive inoculation with a fast-growing non-pathogenic strain can reduce the pathogen load and increase the efficacy of an antibiotic, but only if the antibiotic is used in moderation. Our study shows that including different compartments is essential to create more realistic models of urinary infections, which may help guide new treatment strategies.

## Introduction

Urinary tract infections (UTIs) are a major health problem that affects millions of patients globally every year. Being one of the most common bacterial infections, UTIs present a considerable risk of antibiotic resistance evolution [1–4], and contribute substantially to this global health burden [5]. A large body of research has investigated the course of UTIs both experimentally and clinically to elucidate the infection dynamics as well as the host defense mechanism and the impact of treatment (see e.g. [1, 3, 6–10]). Despite these efforts, recurrent infections are common [3]. Furthermore, UTI treatment is complicated by emerging resistance to multiple antibiotics [4]. A better understanding of UTIs is required to help develop novel approaches such as immunotherapy or prophylactic treatment to overcome these problems [11, 12].

The urinary tract consists of multiple distinct spatial environments for uropathogenic bacteria. In the lumen of the bladder, regular bladder voiding results in frequent clearance, but incomplete voiding is associated with higher infection risks. Consequently, urological voiding restrictions (incontinence, presence of a cystocele, postvoiding residual urine) present strong associations with recurrent UTIs [13, 14]. The bladder epithelium consists of umbrella cells to which bacteria can adhere. Aided by adhesins and pili, bacteria transition from the planktonic phase into the attached phase, from which they may invade into the first layer of epithelial cells in the bladder tissue [1]. Here, the pathogens form biofilm-like intracellular bacterial communities (IBCs), which provide opportunities for growth and protection from the immune system [15].

These three compartments of planktonic, attached, and intracellular bacteria generate a spatial heterogeneity of differing ecological and evolutionary selection pressures. Bladder voiding is able to remove most of the bacteria from the lumen of the bladder, which causes a major population size bottleneck and acts as a protection mechanism against urinary infections [6, 8]. However, its effect on attached and intracellular bacteria is less significant. In contrast, immune responses, e.g. secretion of cytokines, recruitment of neutrophils, and exfoliation driven by mast cells [16, 17], are likely to have a strong effect on the attached and intracellular bacteria. Intracellular bacteria are also affected; their growth rate is reduced and they often assume a coccoid shape [18, 19]. When treated with antibiotics, these bacteria fare much better than their planktonic counterparts [18] due to the reduced drug concentration inside epithelial cells. Such spatial heterogeneity of the antibiotic selection pressure may facilitate resistance evolution in bacteria [20–24]. Therefore, a more efficient treatment of UTIs and the prevention of recurrent UTIs requires a better mechanistic understanding of the role of all these processes on the early dynamics of urinary infections.

In this study, we incorporate both ecological and evolutionary selection pressures into a mathematical model of bacterial population dynamics of planktonic, attached, and intracellular uropathogenic bacteria. The model enables us to investigate the effect of these different compartments on the infection dynamics. We study the effect of accessibility of the attached and intracellular compartment to the invading bacteria. We explore the effect of the intracellular invasion rate and antibiotic permeation in a model in which all compartments can be colonized by the bacteria. We also investigate when colonization resistance by prophylaxis with a non-pathogenic strain (“competitive inoculation”) can limit the spread of a pathogenic infection.

## Materials and methods

**Figure 1:**
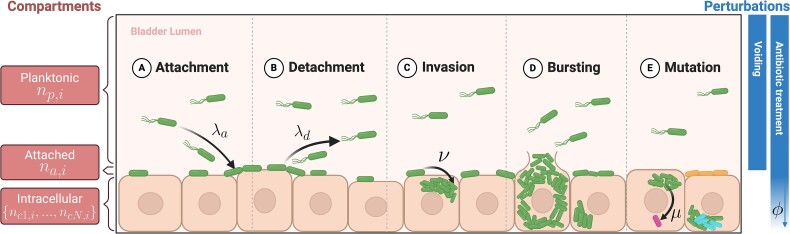
Sketch of the mathematical model of UTIs. Bacterial population in the bladder is divided into three compartments: planktonic, attached, and intracellular, with the number of cells in each compartment denoted by the variables *n_x,j_* [*x* = compartment, *j* = cell type (wild-type, mutants)]. Besides growth and death, bacteria can attach with rate *λ_a_* (A) and detach with rate *λ_d_* (B) from the bladder surface, invade the epithelial cells with rate *ν* (C), forming intracellular colonies and causing the cells to burst (D), which releases bacteria back to the planktonic compartment, and acquire antibiotic-resistant mutations with probability *μ* (E). Resistant mutants result in new lineages, indicated by bacteria with different colors. Furthermore, the population of bacteria is perturbed by bladder voiding and antibiotic treatment. Voiding affects only the planktonic compartment. Antibiotic treatment affects all compartments but to a different degree: the killing probability in the intracellular compartment is a fraction *ϕ ≤* 1 (treatment permeation) of the probability in the planktonic and attached compartments.

We construct a mathematical model to represent the initial dynamics of UTIs within the lumen of the bladder (planktonic bacteria), on the surface (attached bacteria), and inside of the outermost layer of epithelial cells of the bladder wall (intracellular bacteria), see Figs 1 and S1. For computational reasons, we consider only a small patch of 1 mm^2^ of bladder surface and the volume of 10 mm^3^ on top of this surface. This is justified assuming that the bladder surface does not have any large-scale heterogeneities; the results obtained for a small patch can then be extrapolated to the whole bladder. We place *N* = 250 epithelial cells on this surface and inoculate the system with 10^4^ planktonic cells; other compartments are initially empty and become populated as time progresses.

### Stochastic processes in the model

Replication and death are modeled as stochastic Poisson processes. We assume logistic density–dependent birth rates and constant death rates in all compartments. The birth rates *b_i_* and death rates *d_i_* in compartment *i* thus take the form


(1) \begin{equation*} {b}_i={\beta}_i-\left({\beta}_i-{\delta}_i\right)\frac{\sum_j{n}_{i,j}}{K_i}, \end{equation*}

(2) \begin{equation*} {d}_i={\delta}_i, \end{equation*}

with maximum birth rate *β_i_*, intrinsic death rate *δ_i_*, and carrying capacity *K_i_*. Here, *n_i,j_* is the population size of lineage *j* in compartment *i* with *i ∈ {p, a, c*_1_*, . . . , c_N_ }* for planktonic, attached, and the *N* intracellular compartments. We denote the wild-type with *j* = 0 and the mutant lineages with *j* = 1*,*2*, ...* .

Planktonic and attached bacteria replicate with the same maximum birth rate *β_p_* = *β_a_*= 1*.*19 h^*−*1^. We assume the carrying capacity *K_p_* = *K_a_* = 10^6^ cells, which can be justified from experimentally observed bacterial densities (see Supplementary information section 2). We shall also discuss higher planktonic carrying capacities *K_p_* up to 10^7^ cells. For the intracellular population, we assume uncorrelated, normally-distributed birth rates, death rates, and carrying capacities in each epithelial cell. This represents heterogeneity in growth conditions due to differences among the epithelial cells. Mirroring the biofilm-like growth conditions within the epithelial cells, we assume that the average maximum birth rate of intracellular growth equals half of the extracellular maximum birth rate *β_p_*, resulting in an average doubling time of 70 min, in agreement with recent findings [18].

The average death rates are assumed to be equal in all compartments and correspond to 1*/*100 of the planktonic maximum birth rate. We take *K_c_* = 4000 bacteria per cell as the average carrying capacity for intracellular bacteria.

Upon birth, bacteria mutate with probability *μ* = 5 *×* 10^*−*8^. Mutations are neutral in the absence of treatment. Migration between the planktonic and the attached compartment is determined by the attachment rate *λ_a_* = 0*.*2 h^*−*1^ and detachment rate *λ_d_* = 0*.*005 h^*−*1^. Attached bacteria can invade an epithelial cell at rate *ν* (same for all cells). As estimating the invasion rate from the literature is difficult, we will consider a wide range of possible values for this parameter. Once the subpopulation of intracellular bacteria in an epithelial cell reaches 95% of the epithelial cell’s carrying capacity, all bacteria from that subpopulation are released to the planktonic phase, mimicking a burst event similar to fluxing or shedding [9, 18]. After such a burst event, the burst epithelial cell is replaced with an empty cell with identical parameters *β_ci_*, *δ_ci_* and *K_ci_*.

Table 1 summarizes all model parameters; the justification of their values is provided in Supplementary information section 2.

**Table 1:** Reference parameter set. Deviations from these values are reported where applicable.

Symbol	Parameter description	Value
*N*	Number of epithelial cells	250 cells
*K_p_*	Carrying capacity for planktonic bacteria	10^6^ bacteria
*K_a_*	Carrying capacity for attached bacteria	10^6^ bacteria
*K_c_*	Average carrying capacities for intracellular bacteria	4000 bacteria per epithelial cell
*σ_K_*	Standard deviation of intracellular carrying capacity	600 bacteria
*β_p_*	Maximum birth rate of planktonic bacteria	1*.*19 h^*−*1^
*β_a_*	Maximum birth rate of attached bacteria	1*.*19 h^*−*1^
*β_ci_*	Average maximum birth rate of intracellular bacteria	*β_p_*/2 = 0*.*595 h^*−*1^
*σ_β_*	Standard deviation of intracellular max. birth rates	0*.*089 25 h^*−*1^
*δ_p_*	Death rate of planktonic bacteria	*β_p_*/100 = 0*.*0119 h^*−*1^
*δ_a_*	Death rate of attached bacteria	*β_a_*/100 = 0*.*0119 h^*−*1^
*δ_ci_*	Average death rate of intracellular bacteria	*β_ci_* /100 = 0*.*005 95 h^*−*1^
*σ_δ_*	Standard deviation of intracellular death rates	0*.*001785 h^*−*1^
*μ*	Mutation probability per birth	5 *×* 10^*−*8^
*λ_a_*	Attachment rate of planktonic bacteria	0*.*2 h^*−*1^
*λ_d_*	Detachment rate of attached bacteria	0*.*005 h^*−*1^
*ν*	Invasion rate of attached bacteria	Varied, 10^*−*5^.*..* 10^*−*2^ per hour
*κ*	Antibiotic kill-fraction	0*.*999
*ϕ*	Intracellular permeation of treatment	Varied, 0.*..* 1
	First voiding time	4 h
*T*_voiding_	Voiding frequency	Varied, 1.*..* 24 h
*ε*	Voiding fraction	90%
	First treatment time	30 h
*T*_treatment_	Treatment frequency	Varied, 1.*..* 24 h
*n_p_*(0)	Initial number of inoculated bacteria	10^4^ bacteria

### Perturbations

Bladder voiding exerts a cyclic perturbation on the bacteria in the planktonic phase. The typical voiding frequency in humans is every 3–4 h [25]; we explore a larger range of *T*_voiding_ = 1*, ...,* 24 h, with the first voiding at *t* = 4 h post-infection. We also assume that each planktonic bacterium has an *ε* = 90% chance of being removed; this is based on 10% or more residual bladder volume increasing the risk of UTI [26]. Bacteria in other compartments remain unaffected. Antibiotic treatment is modeled as a periodic population bottleneck that removes almost all wild-type bacteria from the planktonic and attached compartments (probability *κ* = 99*.*9% per bacterium, which is within the range determined in [27]). Treatment is first applied at *t* = 30 h post-infection. The fraction of bacteria that is removed from the intracellular compartment is *ϕκ*, where *ϕ* is the drug permeation coefficient. Therefore, drug permeation *ϕ <* 1 can create a refuge for the intracellular bacteria. Values of *ϕ* only slightly less than one already lead to a significant reduction in antibiotic efficiency; for *ϕ* = 0*.*75, only *ϕκ ≈* 75% of intracellular bacteria are removed as compared to 99*.*9% of planktonic bacteria.

### Code and data

We implement the above model as a discrete time stochastic simulation in Python (version 3.10) with a step size of *dt* = 0*.*01 h. The pseudocode in Supplementary information section 1 illustrates the simulation workflow. The simulation code is available on Zenodo at https://zenodo.org/records/13902212 The data files have been deposited at https://zenodo.org/records/13901874.

## Results

To investigate the early dynamics of a urinary infection, we develop a mathematical, stochastic model of a bacterial population in the bladder. Motivated by the differential selection pressures acting on bacteria in the planktonic, attached, and intracellular phases, we define a distinct compartment for each of these three phases (Fig. 1). We further split the intracellular compartment into independent subpopulations for each epithelial cell. Bacteria can migrate between compartments, replicate, potentially creating antibiotic-resistant mutant offspring lineages, and die. All mutants are fully resistant to treatment, but each mutation founds a separate lineage that we track; this helps establish the compartment in which this mutation first occurred. See the Materials and methods section for more details.

### Importance of non-planktonic compartments

**Figure 2:**
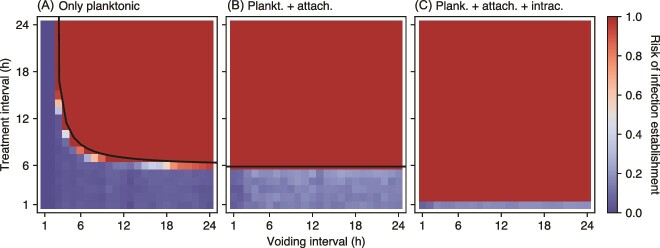
An infection is more likely in the model with planktonic, attached, and intracellular bacteria compared to the model with only planktonic bacteria. (A) Establishment probability of an infection in the model with only planktonic compartment depends on the frequency of bladder voiding and antibiotic treatment. The black line [Equation (3)] represents the boundary between survival and extinction in the deterministic version of the model. (B) Establishment probability in the model with planktonic and attached compartments. The black horizontal line, *T*_treatment_ = *−*(ln(1 *− κ*))*/*(*β_p_ − δ_p_*), is the limit of Equation (3) when *T*_voiding_ *→ ∞*. (C) Establishment probability in the full model, for the invasion rate *ν* = 10^*−*3^. The permeation coefficient *ϕ* = 0*.*75, i.e. treatment of intracellular bacteria is less effective than planktonic/attached bacteria. In each panel, the establishment probability is calculated as the fraction of 1000 replicates with non-extinct bacterial populations at *t* = 100 h. Other parameters are as in Table 1.

We first consider a model with only the planktonic bacteria that will serve as a benchmark for the full model. Voiding, even when assuming an incomplete reduction of the planktonic population, exerts a considerable perturbation and is a very good protection against infection if the pathogenic bacteria do not have access to the attached or intracellular compartments (Fig. 2A). Supplementing this natural defense mechanism with antibiotic treatment further decreases the risk of infection establishment. Similarly, more frequent voiding and shorter treatment intervals lead to a lower risk of infection establishment. Balancing growth and death caused by the antibiotic and periodic voiding leads to the following relationship between the treatment interval *T*_treatment_ and the voiding interval *T*_voiding_, for which the net population growth rate is exactly zero:


(3) \begin{equation*} {T}_{\mathrm{treatment}}=\frac{-\ln \left(1-\kappa \right)}{\left({\beta}_p-{\delta}_p\right){T}_{\mathrm{voiding}}+\ln \left(1-\varepsilon \right)}{T}_{\mathrm{voiding}}, \end{equation*}

where 1 *− κ* is the remaining fraction of bacteria after treatment, 1 *− ε* is the remaining fraction of bacteria after voiding, and *β_p_* and *δ_p_* are the growth and death rates for the planktonic population, respectively. Equation (3) separates the space (*T*_voiding_, *T*_treatment_) in Fig. 2A into two regions: above and below the curve, which correspond to the population surviving/not surviving the combination of voiding and antibiotic treatment. The analytical result agrees very well with our computer simulations (Fig. 2A).

Access to the attached and intracellular compartments, however, largely nullifies the protective effect of voiding and necessitates antibiotic treatment at intervals short enough to prevent regrowth between treatment applications (Fig. 2B and C). The treatment period necessary for the prevention of infection establishment decreases even further if the pathogenic bacteria can access the intracellular compartment and if treatment permeation is imperfect (*ϕ <* 1), thus resulting in an efficient shelter from both voiding and antibiotic treatment.

### Eco-evolutionary dynamics of urinary tract infections

**Figure 3:**
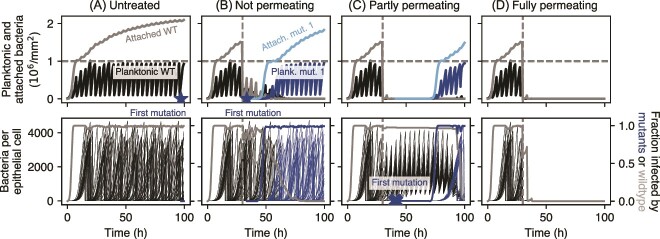
Examples of population dynamics for different intracellular permeability of the antibiotic. (A) No antibiotic. (B) Treatment with an antibiotic that does not permeate into the epithelial cells (*ϕ* = 0). (C) Antibiotic that partially permeates into the epithelial cells (*ϕ* = 0*.*75). (D) Fully permeating antibiotic (*ϕ* = 1). In panels (B–D), treatment is applied every 4 h starting from *t* = 30 h (dashed vertical line), and the invasion rate is *ν* = 10^*−*3^ h^*−*1^. Top row: bacterial density in the planktonic and attached compartments. The carrying capacities *K_p_* and *K_a_* are marked with horizontal dashed lines. Bottom row: number of bacteria within each epithelial cell (thin lines, left vertical axis), and the fraction of infected epithelial cells (thick lines, right axis). Only every 50th intracellular population is shown for clarity. Mutation events are marked by stars (planktonic) and crosses (intracellular compartment). In all cases, *T*_voiding_ = 4 h.

In the following, we consider a scenario in which bacteria can access all three spatial compartments, and investigate its ecological and evolutionary dynamics. If left untreated, bacteria colonize all available compartments of the model, i.e. the planktonic, the attached, and the intracellular one (Fig. 3A). Although voiding initially helps reduce the density in the planktonic phase, planktonic bacteria quickly colonize the epithelial cell surface, which shelters them from voiding. Attached bacteria invade epithelial cells; almost all are infected within hours after inoculation. Bacteria replicate in the cells and when the cells burst, all intracellular bacteria are released to the planktonic compartment. This causes more bacteria to attach to the surface, which increases the attached population beyond the carrying capacity *K_a_*. The burst epithelial cells are replaced with new, bacteria-free epithelial cells leading to a drop in the fraction of infected epithelial cells before new invasion events again increase the fraction of infected cells. Antibiotic-resistant mutations occur in all compartments but are quickly lost in the absence of antibiotic-induced selection due to the stochastic nature of replication, death, and removal processes.

Antibiotic treatment changes these dynamics drastically (Fig. 3B–D), endowing resistant mutants with a fitness benefit (same for all mutants). For non-permeating and not-too-frequent treatment, the intracellular compartment provides a refuge for antibiotic-sensitive bacteria (Fig. 3B), which helps maintaining the wild-type population. This provides a reservoir from which resistant mutants can be generated. However, as these mutants begin to appear, competition with the wild-type population also delays the spread of resistant variants. In contrast, if treatment is fully permeating, the intracellular wild-type population is quickly removed and, in the absence of resistant mutants, the infection is cleared (Fig. 3D). However, if mutants occurred prior to treatment, their large fitness advantage makes a take-over very likely (Fig. S2D). Lastly, for intermediate permeation, the intracellular population is neither cleared nor allowed to grow to large sizes, and cell bursting is rare (Fig. 3C). Therefore, a partially permeating drug or a drug that is ineffective in the intracellular compartment due to other reasons maintains the infection for a long period of time. If a resistant mutant has evolved, it can however co-invade epithelial cells that already harbor wild-type bacteria and outcompete the smaller wild-type population easier than in the case of non-permeating treatment (Fig. 3C).

**Figure 4:**
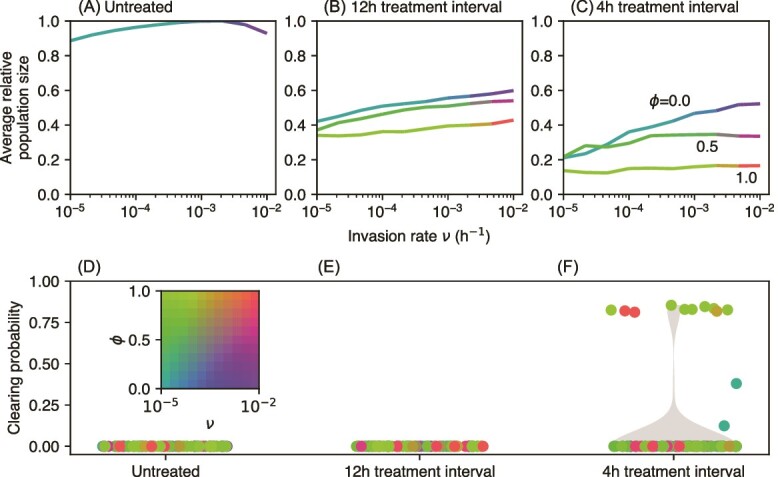
Average population size and infection clearing probability for different invasion rates *ν* and antibiotic permeation *ϕ*. Antibiotic treatment starts at *t* = 30 h and is repeated every 12 h in panels (B) and (E), and every 4 h in panels (C) and (F). Panels (A) and (D) show the results in the absence of treatment. Average population size is shown as a fraction of the total carrying capacity ${K}_p+{K}_a+\sum_i{K}_{ci}$, averaged over the time period *t* = 80*...*100 h. clearing probability is measured as the fraction of replicates in which the bacterial population is extinct at *t* = 100 h. The inset in the bottom row maps the color of the points to combinations of *ν* and *ϕ*. We simulated 1000 replicates for each combination (*ν, ϕ*).

So far (Fig. 3), we have considered only three values of the permeation coefficient *ϕ* and one value of the invasion rate *ν*. By exploring a range of values of *ν* and *ϕ*, we find that the qualitative outcome depends very little on these parameters, unless the antibiotic is very permeable or the invasion rate is very low (Fig. 4). The probability for clearing the infection is non-zero not only for frequent treatment with a high drug permeation but also for low permeation and low invasion rate (Fig. 4D–F). In the latter case, planktonic and attached bacterial populations are suppressed by treatment and voiding. As the invasion rate is low, only a few epithelial cells get infected. Intracellular bacteria grow mostly unaffected, because the antibiotic permeation is low. The host cells soon burst, releasing bacteria to the planktonic compartment, from which they are efficiently removed.

### Lineage tracing elucidates the origin of resistant mutants

Our stochastic simulations allow us to trace resistance evolution from the emergence of mutants to their eventual spread or extinction. Although resistant mutants always emerge in a large enough wild-type population with a high replication rate, their survival and eventual dominance over the wild-type requires a selection for resistance, such as the one imposed by antibiotic treatment. In the absence of selection, most such mutants (but not all) are lost to genetic drift. The three compartments of our model (planktonic, attached, and internalized) represent the distinct ecological niches that bacteria face in UTIs. We ask which of these niches contributes most mutants that eventually fix in the population.

**Figure 5:**
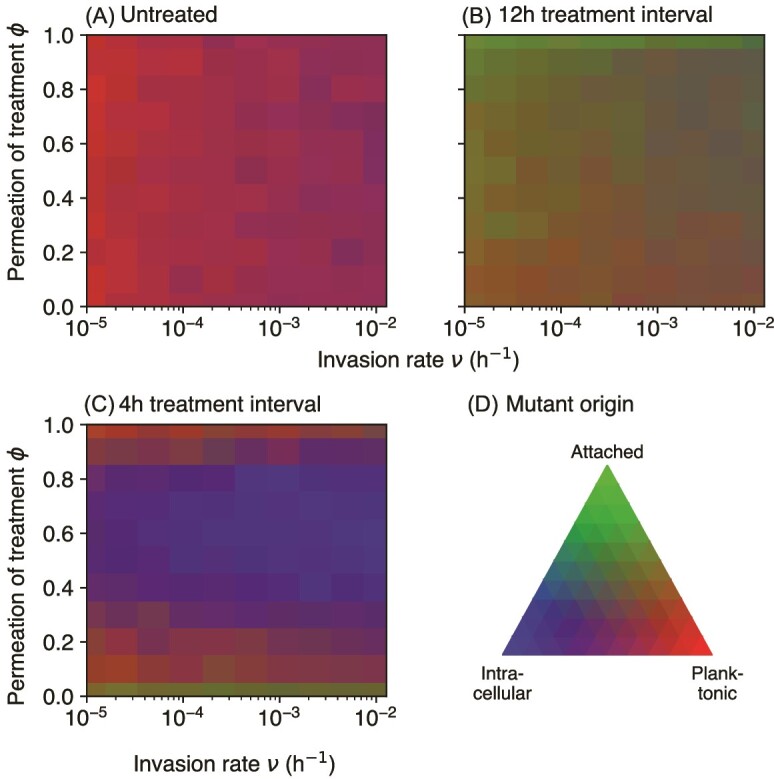
Origin of surviving mutants. Panels (A)–(C) shows where the mutants came from (planktonic, attached, or intracellular compartment) using a mix of three colors, for different invasion rates *ν* and treatment permeations *ϕ*. The colors are linearly mixed in proportion to the frequency of mutants coming from a particular compartment, illustrated in panel (D). Panels (A)–(C) represent three treatment levels: untreated, treatment every 12 h, and treatment every 4 h (same as in Fig. 4). In each case, we simulated 1000 replicates.

We find that when all compartments are of the same size (${K}_p={K}_a={K}_c={10}^6$), the origin of successful mutants depends on the strength of antibiotic treatment (Fig. 5). In the case of no treatment, most successful resistant mutants come from the planktonic phase, with some contributions from the intracellular phase for high invasion rates (Fig. 5A). Low–frequency antibiotic treatment shifts the balance toward the attached phase (Fig. 5B), whereas for high–frequency antibiotic treatment, the intracellular phase dominates, especially for intermediate permeation (Fig. 5C). These results can be understood by looking at the population sizes and mutation rates in each compartment (Figs S3 and S4). In the absence of treatment, voiding leads to a larger attached population than the planktonic and intracellular populations (Fig. S3A), but the high density of the attached phase prohibits replication. This leads to a positive correlation between average population size and the number of mutations only for the planktonic and intracellular compartments (Fig. S5). Most mutants therefore originate in the planktonic phase for low invasion rates ν, with a contribution from the intracellular phase for large ν (Fig. S4A).

Infrequent treatment, in combination with voiding, strongly reduces the wild-type population in all compartments and gives the mutants a selective advantage for not-too-small permeation parameter *ϕ* (Fig. S3B). Similarly to the no-treatment scenario, mutations occur predominantly in the planktonic compartment, but voiding eliminates most of them (Fig. S4B, upper panel). However, reduced population size in the attached compartment now allows for replication and a moderate number of mutations also in this compartment (Fig. S4B, lower panel). This now causes the attached compartment to contribute successful mutants (cf. mixed red and green colors in Fig. 5B).

Frequent treatment alters the balance between wild-type and mutant bacteria (Fig. S3C). Mutants reach high average population sizes in the attached compartment for low and high permeation, but not for intermediate values, where wild-type cells dominate the intracellular compartment. Slower growth at these intermediate values of permeation prevents bursting of the epithelial cells and the expulsion of intracellular bacteria. Sustained intracellular wild-type populations compete with the mutants and slow down their take-over. Nevertheless, intracellular populations are replication hot-spots which give rise to many mutations whose survival probability, however, is often lower than those from the attached compartment (Fig. S4C).

The dominant origin of mutations changes when the compartments have different sizes. In particular, if the planktonic compartment is much larger (*K_p_* = 10^7^ cells) compared to the two other compartments, most successful mutations come from the planktonic phase (Fig. S11). However, this scenario requires a very high density of bacteria in urine (cf. Supplementary information section 2). For a more plausible scenario with *K_p_* = 3 *×* 10^6^, the results are more in line with those discussed above for equally-sized compartments (Fig. S12).

### Competitive inoculation with a non-pathogenic strain helps prevent infections

We have shown that the competition between wild-type and resistant mutants is an important determinant of infection dynamics and resistance evolution. In light of the risk of antibiotic treatment failure due to resistance evolution it is imperative that alternative or supplementary treatment types are developed against UTIs. Besides treating an acute infection, the prevention of the initial establishment of pathogenic bacteria in the urinary tract is an important aim to reduce the overall disease burden and increase the well-being of many patients affected by recurrent UTIs. One such approach is the prophylactic or adjuvant introduction of non-pathogenic bacteria into the urinary tract, i.e. competitive inoculation, to prevent the establishment of a more pathogenic strain. In the following, we will explore whether non-pathogenic bacterial strains that induce an asymptomatic bacteriuria (labeled ABU hereafter) could be used to prevent the spread of pathogenic strains (labeled PATH) via competition, and whether such treatment could also limit the evolution of resistance to a parallel antibiotic treatment.

**Figure 6:**
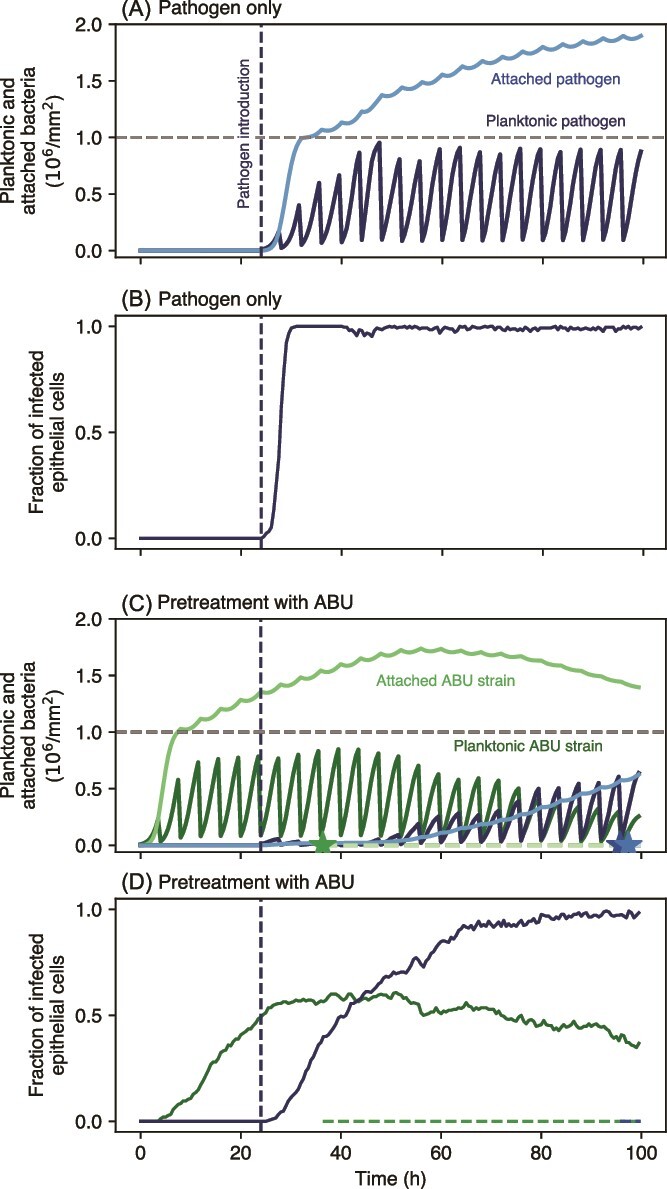
Population dynamics of pathogenic bacteria without (A and B) or with (C and D) competitive inoculation with an ABU strain. A total of 10^4^ pathogenic PATH bacteria are introduced at *t* = 24 h into the planktonic compartment. For the pre-treated case, 10^4^ non-pathogenic ABU bacteria are also introduced at *t* = 0 h. We assume the invasion rate of the PATH strain to be *ν*_PATH_ = 10^*−*3^ per hour, which is two orders of magnitude higher than the invasion rate of the ABU strain *ν*_ABU_ = 10^*−*5^ per hour. The pathogen birth rate is assumed to be 10% below the growth rate of the non-pathogenic ABU strain (*β*_ABU_ = 1*.*19 h^*−*1^, *β*_PATH_ = 1*.*07 h^*−*1^). No antibiotic treatment is applied. The voiding interval is 4 h. As in Fig. 3, stars mark mutation events in the planktonic compartment, which in this replicate simulation occur only in the pre-treated scenario.

**Figure 7:**
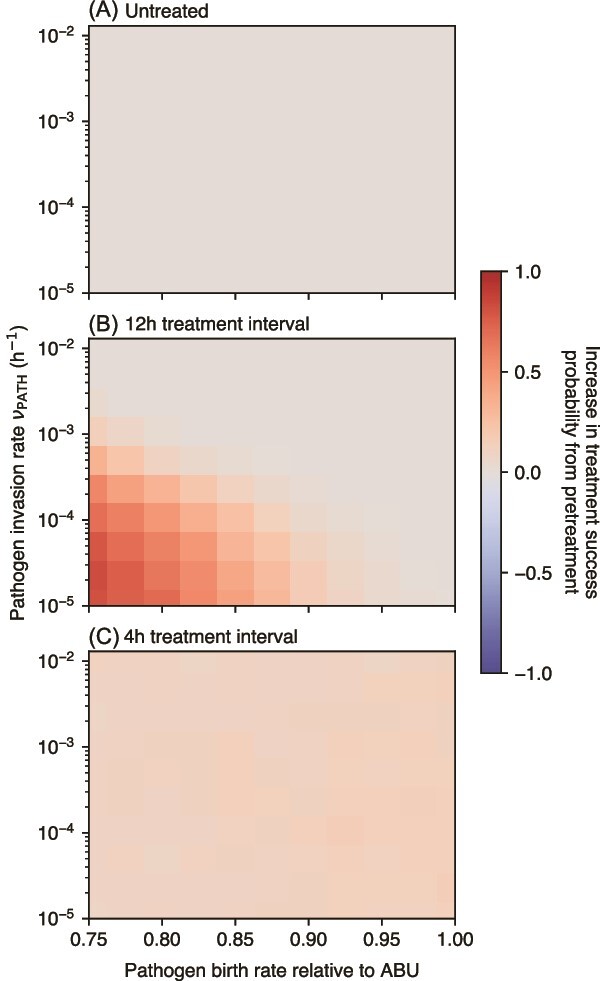
Effect of prophylactic competitive inoculation on the extinction probability of the pathogen for different antibiotic treatment intervals. A larger probability of clearing the pathogen is indicated by a positive value. As in Fig. 4, pathogen extinction is determined at *t* = 100 h after adding the pathogen at *t* = 24 h. Antibiotic treatment here is fully permeating (*ϕ* = 1).

We assume that the increased pathogenicity of the PATH strain would manifest as a higher invasion rate *ν*, at the cost of a decreased birth rate relative to the ABU strain. For the ABU strain, we thus assume an invasion rate at the lower end of the parameter range that was investigated above, but a faster birth rate than the PATH strain. This trade-off causes that competitive inoculation with an ABU strain delays the spread of the PATH strain (Fig. 6). This delay leads to a decreased pathogen population (Fig. S6), in particular in the case of no antibiotic treatment, and predominantly in the attached compartment. Antibiotic treatment decreases this effect considerably as treatment suppresses also the ABU strain, thus weakening its competitive suppression of the PATH strain. However, in combination with antibiotic treatment, ABU strain pre-treatment is able to increase the probability of eradicating the pathogen (Fig. 7). In the case of infrequent antibiotic treatment, successful treatment is possible for a considerably lower pathogen birth rate as well as an invasion rate only slightly higher than the ABU strain (Fig. 7B). For frequent treatment, the increase of treatment success probability is more moderate, but independent of pathogen growth and invasion rates (Fig. 7C).

Competitive inoculation decreases the effective mutation rate of the pathogen (Fig. S7), but may actually increase the total number of mutations arising from both ABU and PATH bacteria due to a higher overall abundance of bacteria (Fig. S8). For low pathogen invasion and birth rates, pre-treatment with ABU bacteria tends to reduce the survival of resistant mutants from the PATH background, but increases it for weaker trade-off, i.e. only slight reduction of pathogen birth rate and much higher pathogen invasion rate (Fig. S9). A similar pattern is apparent when considering the survival of resistant mutants from both PATH and ABU backgrounds (Fig. S10). These findings indicate the potential of unintentionally aggravating the problem of resistance evolution by prophylactic pre-treatment.

## Discussion

After a prior UTI by *Escherichia coli*, 20%*–*30% of women suffer a recurrent UTI within the next 6 months [28]. Besides reinfection from external sources or the intestinal reservoir, persistence within the urinary tract is thought to be one of the sources of these recurrent infections [29]. IBCs are a possible cause for this persistence [3, 15, 30]. We have presented here a mathematical model of the initial phase of UTIs that captures the dynamics of IBCs in addition to planktonic bacteria in the bladder lumen and bacteria attached to the urothelium. We have shown that access to the non-planktonic compartments, the invasion rate into the intracellular compartment, and the vulnerability of this potential shelter to antibiotic treatment (defined by treatment permeation *ϕ*) are decisive parameters for the eco-evolutionary dynamics of early UTIs.

The different spatial compartments provide distinct growth and perturbation conditions. The planktonic compartment of bacteria is vulnerable both to bladder voiding and antibiotic treatment. The attached compartment is sheltered against voiding but still susceptible to antibiotic treatment. The intracellular compartment may provide shelter against both perturbations, unless a well-permeating antibiotic is used. Transitioning between these compartments thus allows the bacterial population to moderate the differing selection pressures and can generate intricate ecological and evolutionary infection dynamics, which may explain the difficulty of preventing recurrent UTIs.

We observe a trade-off between the chance of eradicating the infection and the risk of resistance evolution. More aggressive treatment, i.e. shorter treatment interval or higher permeation, exerts not only a stronger perturbation but also a higher selection pressure for resistant mutants, thus increasing their survival probability. This general trade-off is well-known and forms the basis for evolution-informed treatment approaches for bacterial infections and cancer [31–34]. These approaches recommend lower treatment-induced selection pressures to delay treatment failure when resistance is already present or when it would inevitably evolve. Our study shows that spatial heterogeneity and refuge from treatment may further complicate the search for the most efficient balance between infection eradication and the risk of resistance evolution.

We find that intermediate antibiotic permeation can result in long-term, intracellular bacterial populations, similar to persistent IBCs or the recalcitrating effect of biofilms [24]. These intracellular subpopulations can act as mutational hotspots due to the large population size and the high number of replications. Non-perfect antibiotic permeation together with the high treatment frequency prevent bursting of the epithelial cells without eradicating the intracellular populations. This could undermine parts of the natural defense against infections; our results indicate that accounting for the spatial distribution of perturbations is necessary to better understand the eco-evolutionary dynamics in UTIs. Our results show that wild-type population size is a good predictor for the occurrence of mutations, but only in the planktonic and internalized compartment, as attachment flux and shelter from voiding can create attached population sizes beyond the carrying capacity, resulting in the lack of replication. Furthermore, population size is a bad predictor for the survival probability of mutants. Here, the compartment of origin is decisive. Mutants that arise in the attached compartment are more likely to survive than planktonic mutants. Survival of intracellular mutants depends on high antibiotic selection pressure, i.e. short treatment intervals and high permeation.

One important consequence of the availability of the intracellular compartment is that the planktonic population size is a bad indicator of an ongoing infection. We show that many combinations of voiding and antibiotic treatment intervals lead to small planktonic population sizes but the attached and intracellular populations remain large. As the presence of bacteria in urine (represented by our planktonic compartment) is a routine diagnostic tool for acute UTIs, this poses a serious risk of terminating antibiotic treatment before the infection has been cleared, elevating the risk of antibiotic misuse and widespread resistance evolution. Our findings support recent advocacy for using other methods to detect UTIs, such as molecular diagnostic methods [35, 36], which might be particularly important for patients with chronic lower urinary tract symptoms [37].

We also find that prophylactic competitive inoculation with a non-pathogenic strain can hinder the establishment of a pathogenic urinary infection, which is supported by clinical evidence [38–40]. For infrequent antibiotic treatment, this positive effect depends on smaller invasion benefits of the pathogen, and it becomes more prominent for a larger growth advantage of the non-pathogenic strain over the pathogenic one. Indeed, non-pathogenic candidate strains are often found to grow faster than pathogenic strains [41, 42]. However, competitive inoculation is less beneficial for frequent antibiotic treatment, which is reasonable given our assumption of the antibiotic treatment also targeting the non-pathogenic strain. Judging whether this interaction is also present in patients requires further research.

Our model—as any idealized mathematical model—has many limitations. We have listed some of them below:

*Biological realism.* All processes in the model are highly simplified: replication is Poissonian, voiding occurs at regular intervals, the antibiotic always kills the same fraction of cells. The carrying capacities are fixed and do not vary in time as the bladder expands. The immune system is not accounted for.

*Computational complexity.* Only a small patch of the bladder is modeled, and the assumed mutation probability is at the upper end of what would be expected for most antibiotics for non-mutator strains. This is necessary because a stochastic simulation of millions of intracellular clusters of bacteria that could reside in the bladder is not computationally feasible.

*Parameters.* Although we tried to provide realistic estimates for all parameters, this was not always possible due to the lack of adequate experimental data. Moreover, many parameters are likely to vary significantly from infection to infection; hence, it may not be possible to converge on a single set of parameters representative for all UTIs.

Nevertheless, the limitations discussed above do not impede the qualitative analysis of the relative importance of various ecological and evolutionary factors presented in this work.

One final limitation of our study is the focus on the dynamics of early UTIs. Important aspects at later stages include cell-based immune responses, changes of bacterial phenotypes, e.g. filamentation or biofilm formation in the attached phase, and the invasion into deeper layers of the bladder tissue [3, 43]. Although these aspects are certainly important to understand the course of the disease, our focus on the initial stages of UTIs aims at supporting the early resolution and prevention of this frequent infection and thus may contribute to improved health and quality of life for millions of patients.

## Supplementary Material

Supplementary_Information_+_Supplementary_Figures_wrae191

## Data Availability

All Python scripts used in this study have been deposited on Zenodo at https://zenodo.org/records/13902212. The simulation outputs can be found at https://zenodo.org/records/13901874.
