# Supplementary material for: Pathogen non-planktonic phases within the urinary tract impact early infection and resistance evolution
Source: ISME J. 2024 Sep 26;18(1):wrae191. doi: 10.1093/ismejo/wrae191 (PMC11499890; doi:10.1093/ismejo/wrae191)
Supplement: Supplementary_Information_+_Supplementary_Figures_wrae191 [file supplementary_information_+_supplementary_figures_wrae191.docx]

**Pathogen non-planktonic phases within the urinary tract impact early infection and resistance evolution**

Supplementary Information

Michael Raatz1, Amanda de Azevedo-Lopes1, Karolina Drabik2, Arne Traulsen1, and Bartlomiej Waclaw2,3

1Department of Theoretical Biology, Max Planck Institute for Evolutionary Biology, Plön, Germany

2Dioscuri Centre for Physics and Chemistry of Bacteria, Institute of Physical Chemistry (IChF), Polish Academy of Sciences, Warsaw, Poland

3School of Physics and Astronomy, University of Edinburgh, Edinburgh, United Kingdom

September 25, 2024

# 1 Computer algorithm

The following pseudo-code represents the algorithm we use to simulate the model.

**while**  ${t<t}_{max}$ **do**

**for** *lineage* $j\in\{0, 1, . . . \}$ **do**

**Attachment and detachment dynamics**

determine the number of attaching bacteria from $\mathrm{Binom}\left( n_{p,j}\left( t \right), \lambda_{a}\left( 1-\frac{n_{a}\left( t \right)}{n_{a}\left( t \right)+K_{a}} \right)dt \right);$

**end**

determine the number of detaching bacteria from $\mathrm{Binom}(n_{a,j}\left( t \right),\lambda_{d}\mathrm{dt})$ ;

resolve attachment and detachment dynamics;

**Invasion dynamics**

draw number of newly infecting bacteria from $\mathrm{Binom}\left( n_{a,j}\left( t \right), \nu dt \right);$

randomly distribute the infecting bacteria among the epithelial cells;

**Growth dynamics**

draw the number of births $B_{i,j}$ in each compartment *i* from Poisson distribution with mean $b_{i}n_{i,j}dt$;

draw the number of deaths in each compartment *i* from Poisson distribution with mean $d_{i}n_{i,j}dt$;

determine the number of mutants among the births from $\mathrm{Binom}\left( B_{i,j}, \mu\right);$

advance population sizes accounting for births, deaths, and mutations, thus creating $n_{i,j}\left( t+dt \right)$

**Potential voiding** (if *t* advances beyond the next predefined voiding interval)

**if** *voiding* **then**

determine the remaining planktonic population size of each lineage $n_{p,j}\left( t+dt \right)$from $\mathrm{Binom}\left( n_{p,j}\left( t+dt \right), 0.1 \right)$

**end**

**Potential treatment** (if *t* advances beyond the next predefined treatment interval)

**if** *treatment* **then**

**for** *lineage* $j \{0, 1, . . . \}$ **do**

Determine the remaining population sizes after treatment accounting for potentially reduced permeation

**end**

**end**

$n_{p,j}\left( t+dt \right)\sim\mathrm{Binom}\left( n_{p,j}\left( t+dt \right), (1-\kappa) \right)$

$$n_{a,j}\left( t+dt \right)\sim\mathrm{Binom}\left( n_{a,j}\left( t+dt \right), \left( 1-\kappa\right) \right)$$

$n_{c1,j}\left( t+dt \right)\sim\mathrm{Binom}\left( n_{c1,j}\left( t+dt \right), (1-\phi\kappa) \right)$

$\vdots$

$n_{cN,j}\left( t+dt \right)\sim\mathrm{Binom}\left( n_{cN,j}\left( t+dt \right), (1-\phi\kappa) \right)$

**Potential bursting for** *i ∈ {c*1*, . . . , cN}* **do**

**if** $\sum_{j} n_{i,j}>0.95 K_{i}$**then**

move all bacteria from cell *i* to the planktonic pool;

reset the epithelial cell as empty

**end**

**end**

**end**

*t* = *t* + d*t*

# 2 Justification of the parameters of the model

In this section, we briefly discuss our choices of the parameters. All numerical values of the parameters have been summarized in Table 1 in the main text.

*Bladder surface and volume.* Assuming that the bladder surface does not have any large-scale heterogeneities, we consider only a small patch of 1 mm^2^ of bladder surface and the volume of 10 mm^3^ on top of this surface. This stems from the bladder volume of hundreds of ml divided by its surface area in mm2. For example, a 400 ml spherical bladder has a surface area of 26 000 mm2, hence the average height of the column of urine above a 1 mm2 patch is about 15 mm. We take a slightly smaller value of 10 mm to account for fluctuations of the bladder volume throughout the day.

*Number of bladder epithelial cells.* We estimate the average area and height of epithelial cells using images from [[1](#_bookmark3), [2](#_bookmark4)]; this gives the average area of 4000 µm^2^ and average height of 4 µm. We then divide the area of the simulated bladder surface (1 mm^2^) by the average area occupied by an epithelial cell to

obtain the number of epithelial cells in the 1 mm2 patch.

*Carrying capacity of the planktonic compartment.* For the planktonic phase, we assume that the stationary density of bacteria in urine is 10^8^ mL*^−^*^1^ (within the range of Ref. [[3](#_bookmark5)]). This is at the lower end of the spectrum of reported densities (between 10^8^ and 10^9^ mL*^−^*^1^ found in *in vitro* stationary cultures [[4](#_bookmark6)]); Ref. [[5](#_bookmark7)] reports densities as high as 10^9^ mL*^−^*^1^. The assumed maximum density of 10^8^ mL*^−^*^1^ leads to the carrying capacity for birth of *K_p_* =10^6^ cells.

*Carrying capacity of the attached compartment.* Assuming that attached bacteria form a closely- packed monolayer and require approximately 1 µm^2^ per cell, the carrying capacity for birth in the attached phase is *K_a_* = 10^6^ cells in the 1 mm^2^ surface patch.

*Carrying capacity of the intracellular compartment.* We assume that the maximum IBC volume is 4000 µm^3^ (upper range of Ref. [[6](#_bookmark8)]). Considering a typical UPEC bacterial volume of 1 µm^3^, we estimate the average carrying capacity per epithelial cell to be *K_c_* = 4000 bacteria. This choice, together with the number of epithelial cells *N* = 250, makes the total carrying capacity of the intracellular compartment the same as the planktonic and attached compartments (10^6^ bacteria), which is convenient for studying

the origin of the mutants.

*Attachment rate.* We calculate the adhesion rate from *in vitro* experiments which used UPEC bacteria and epithelial bladder cells [[7](#_bookmark9)]. Figure 5a from that reference shows that about 30% bacteria adhered to epithelial cells after 1 h of incubation (without centrifugation). Assuming that attachment occurred with a constant rate *λ_a_*, we can estimate *λ_a_* from the fraction of adhered bacteria as $f= 1- e^{-\lambda_{a}t}$, where $f$ = 0*.*3 and *t* = 1 h. This gives *λ_a_ ≈* 0*.*35 h*−*1. As this value may still be on the upper end of the scale as it has been measured in the absence of any processes that would interfere with adhesion (urine sloshing, bladder expansion and contraction), we use a slightly lower value *λ_a_* = 0*.*2 h*−*1_._

*Detachment rate.* Due to the lack of adequate experimental measurements of the detachment rate

of UPEC attached to bladder epithelial cells, we use a value from Ref. [[8](#_bookmark10)], which describes a model of biofilm colonization of abiotic surfaces. However, the exact value of *λ_d_* is not critical as long as it is much lower than the attachment rate *λ_a_*. In particular, we have checked that Fig. 5 is not affected if

we assume *λ_d_* = 0 (Fig. S13).

*Invasion rate.* References [[9](#_bookmark11), [10](#_bookmark12)] report invasion rates on the order of 10*^−^*^2^ h*^−^*^1^. For example, Ref.

[[10](#_bookmark12)] shows that 33% of cells were invaded by bacteria after 20 h; this gives the invasion rate of about $(1/3)/ 20 h^{-1} \approx1/60 h^{-1}$. We use this value as the upper limit for the invasion rate *ν*, and explore a range of values *ν* = 10*^−^*^5^*, ... ,* 10*^−^*^2^ per hour.

*Mutation probability*. We assume that bacteria mutate with probability *µ* = 5 *×* 10*^−^*^8^ upon birth. This value is based on the typical mutation rate of UTI *E. coli* isolates to the antibiotic rifampicin

determined in Ref. [[11](#_bookmark13)].

*Birth and death rates.* References [[4](#_bookmark6), [12](#_bookmark14)], report a doubling time of 40 *±* 20 min for extracellular bacteria in urine. We consider the minimum doubling time of 35 min, which corresponds to a maximum birth rate of extracellular bacteria, *β_p_* = *β_a_* = ln(2)*/*35 min = 1*.*19 h*^−^*^1^. For the intracellular bacteria, [[13](#_bookmark15)], Figure 4D, reports a doubling time of 70 min, which corresponds to *β_ci_* = 0*.*595 h*^−^*^1^ = *β_p_/*2. This rate has been measured by first purging planktonic cells with ampicillin, which may have also affected intracellular bacteria, thus the pre-treatment growth rate may be faster than assumed. All death rates are set to be 1% of the maximum birth rate *β_p_*. This reflects the fact that bacteria rarely die if not subject to external stress (e.g. antibiotics).

# 3 Supplementary Figures


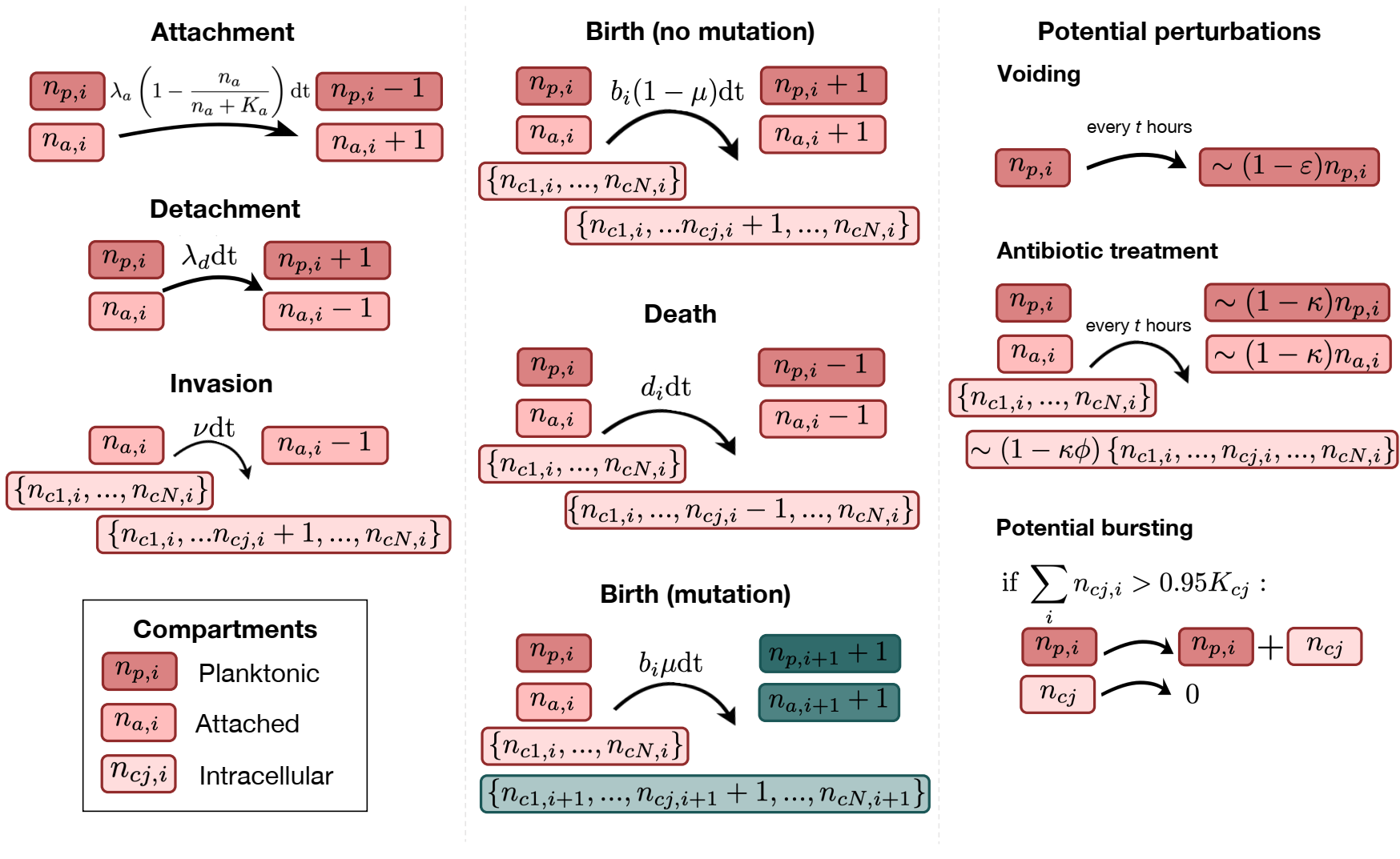


Figure S1: **Graphical representation of all processes that occur in the model.** Definitions of all parameters and their values can be found in Table 1. Mathematical expression above the arrows denote the probability with which a given process occurs during a small time step dt. The algorithm used to simulate the model can be found in Sec. Computer algorithm.


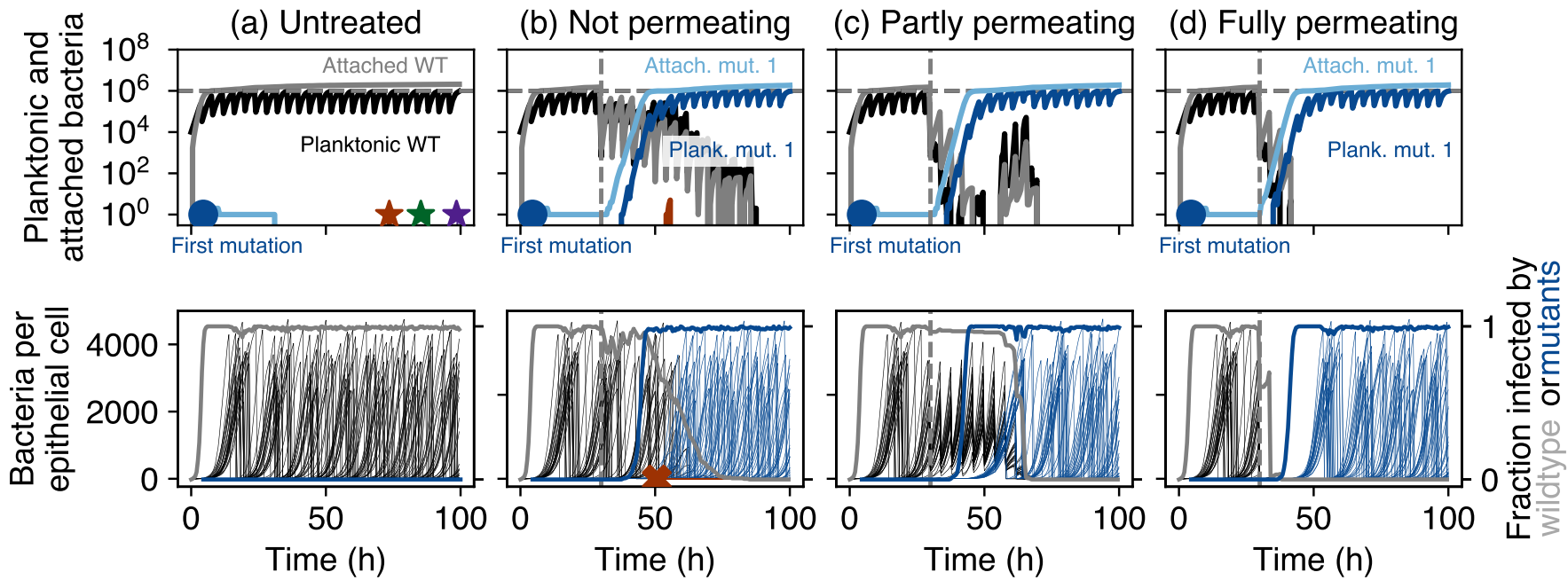
Figure S2: **A different run of the model for the same sets of parameters as in Fig. 3.** Due to the stochastic nature of the model, population dynamics differs to that of Fig. 3. (a) No antibiotic. (b) Treatment with an antibiotic that does not permeate into the epithelial cells (*ϕ* = 0). (c) Antibiotic that partially permeates into the epithelial cells (*ϕ* = 0*.*75). (d) Fully permeating antibiotic (*ϕ* = 1). Mutation events are marked by circles (attached), stars (planktonic) and crosses (intracellular compartment). In contrast to Fig. 3d, in panel (d) the mutants rapidly take-over the population due to a large fitness advantage.


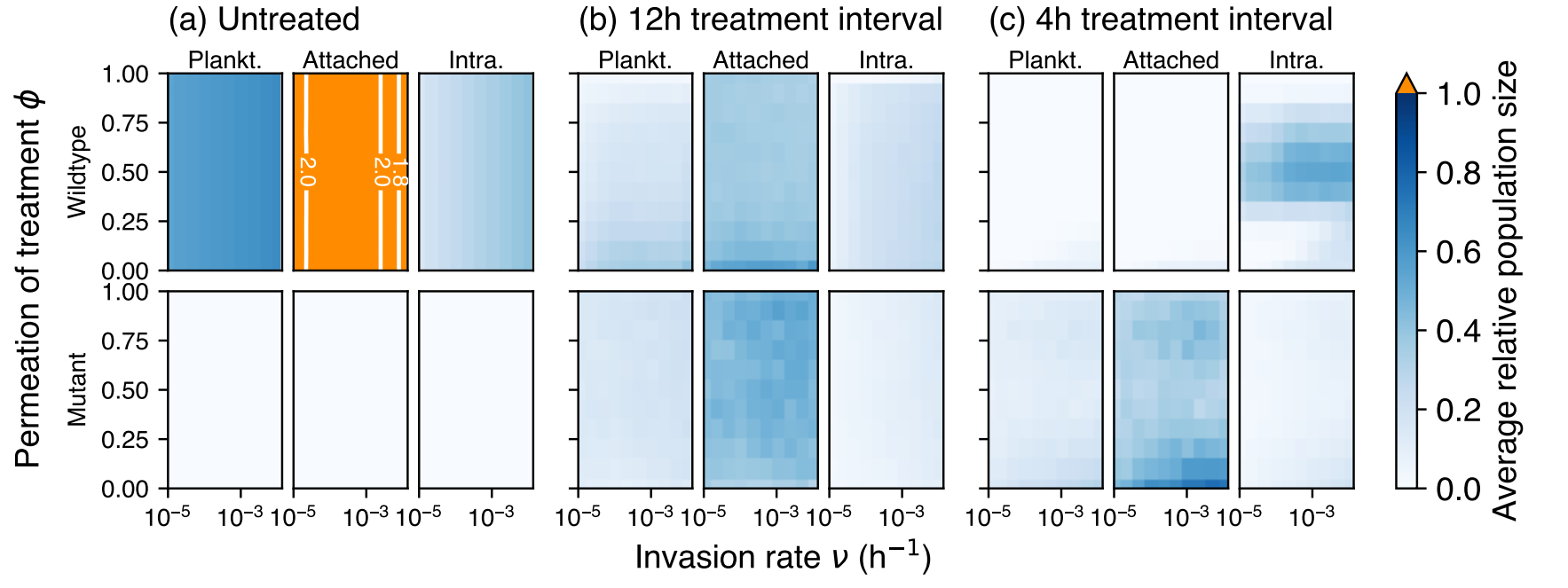


Figure S3: **Average relative population size in each of the three compartments.** The average population size of wildtype (top row) and mutant (bottom row) across 1000 replicates is plotted relative to the carrying capacity of the respective compartment, i.e. *K_p_*, *K_a_* and $\sum_{i} K_{ci}$ for planktonic, attached and intracellular, respectively. The orange colouring indicates population sizes beyond the carrying capacity of the respective compartment with the white lines showing isoclines for orientation. Simulations have been performed as described in Fig. 4.


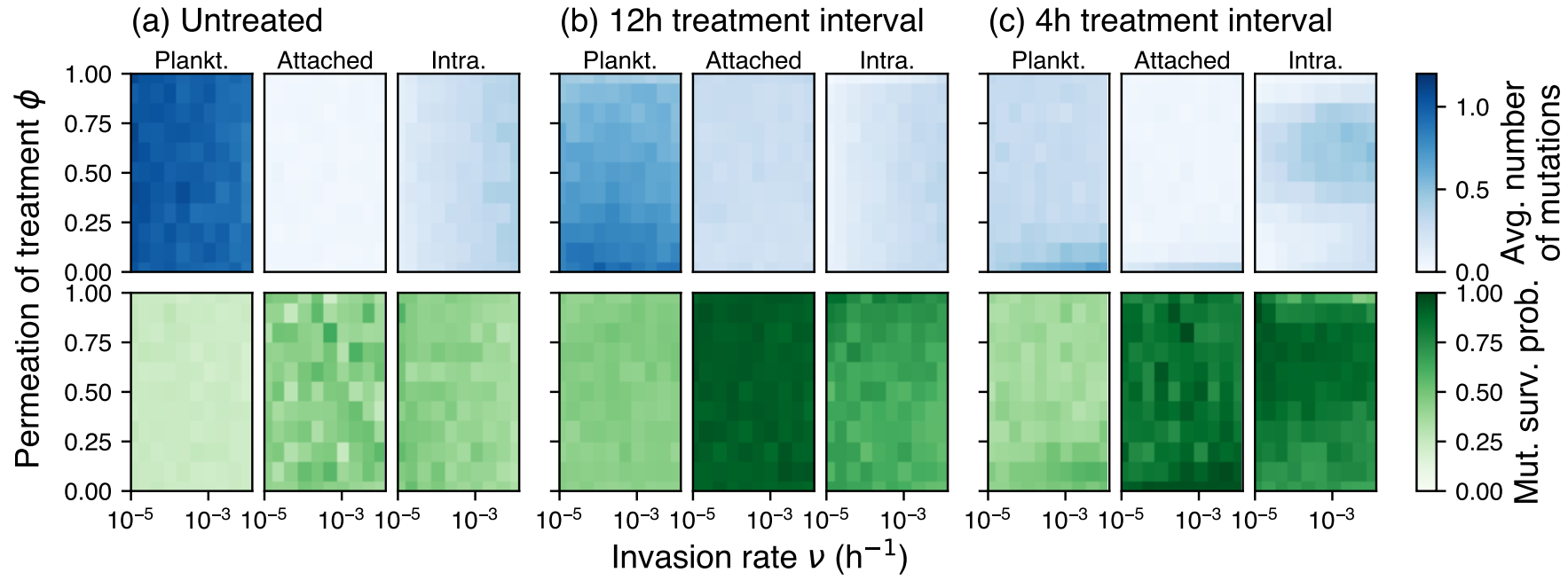


Figure S4: **The observed number of mutations and survival probability of mutants for different treatment types in each of the three compartments.** Top row: Observed average number of mutations in the respective compartment across 1000 replicates during 100 h with treatment start at *t* = 30 h. Bottom row: Probability of survival for these mutants during the observation period. Simulations have been performed as described in Fig. 4.


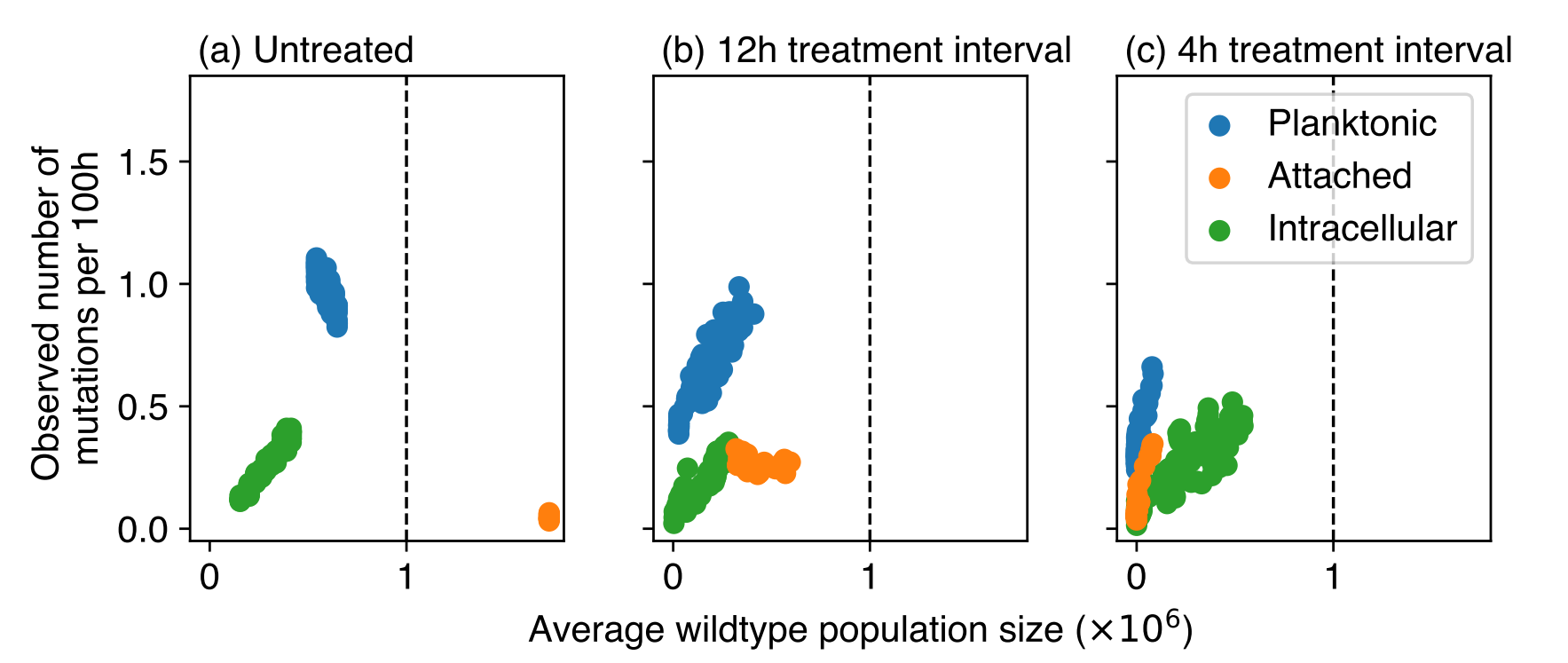


Figure S5: **Correlation of observed mutation rate (number of observed mutations per 100 h) and average wildtype population size in the different antibiotic perturbation scenarios.** The points correspond to the different combinations of invasion rate *ν* and treatment permeation *ϕ* explored in Fig. 4. Colours denote different compartments. The dashed vertical line shows the carrying capacities for birth (assumed to be equal in all compartments). For the intracellular compartment, *K_c_* is shown.


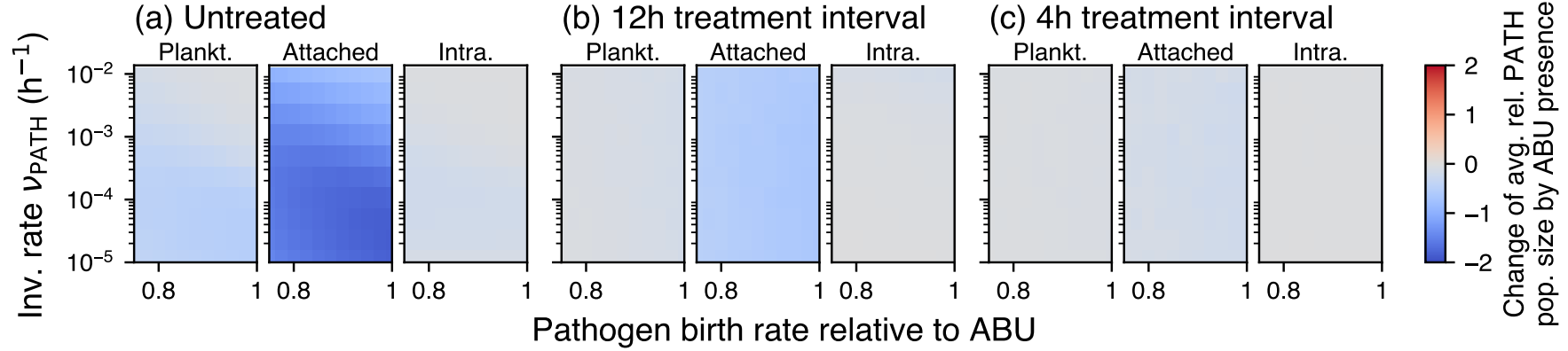


Figure S6: **Effect of prophylactic pretreatment on pathogen population size for different antibiotic treatment intervals.** The colour gradient shows the change in the relative pathogen population size by compartment averaged over 1000 replicates for *t* = 80 *. . .* 100 h. Negative values (blue) indicate a lower average pathogen population size when prophylactic treatment is added at *t* = 0 h, compared to no prophylaxis. As in Fig. 6, the pathogen is added at 24 h.


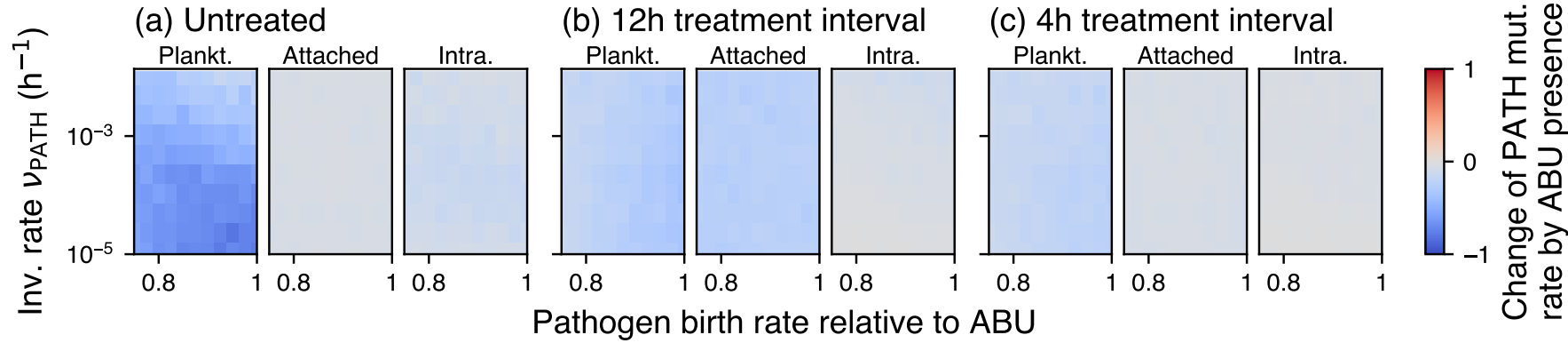


Figure S7: **Effect of prophylactic** **pretreatment on the observed pathogen mutation rate.** Negative values indicate a lower mutation rate (no. of mutants generated per 100 h) on the pathogen background if pretreated with a non-pathogenic strain.


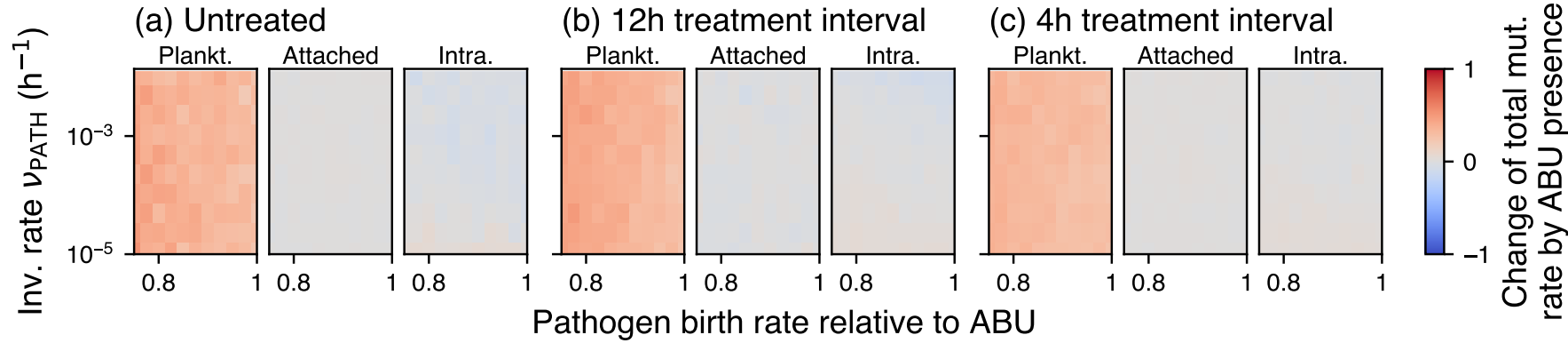


Figure S8: **Effect of prophylactic pretreatment on the observed total mutation rate (including mutations on ABU and PATH background).** In contrast to Fig. S7, all mutations are counted here.


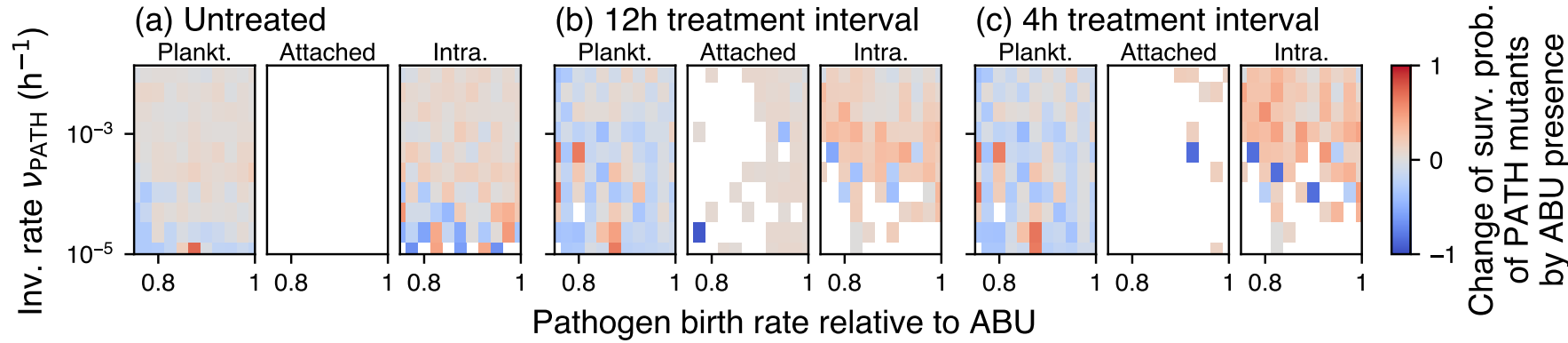


Figure S9: **Effect of prophylactic pretreatment on the survival probability of pathogen mutants.** Negative values indicate a lower establishment probability of pathogenic mutants because of prophylactic pretreatment. Blank values indicate parameter combinations for which mutants did not appear in both scenarios (with and without ABU).


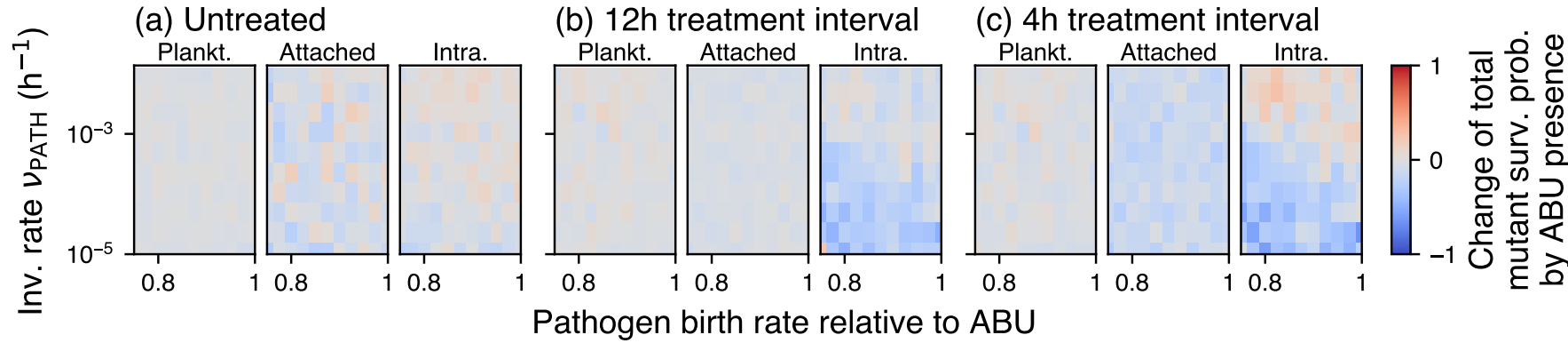


Figure S10: **Effect of prophylactic pretreatment on the survival of either non-pathogen or pathogen mutants.** Negative values indicate a lower establishment probability of mutants from all backgrounds because of prophylactic pretreatment.


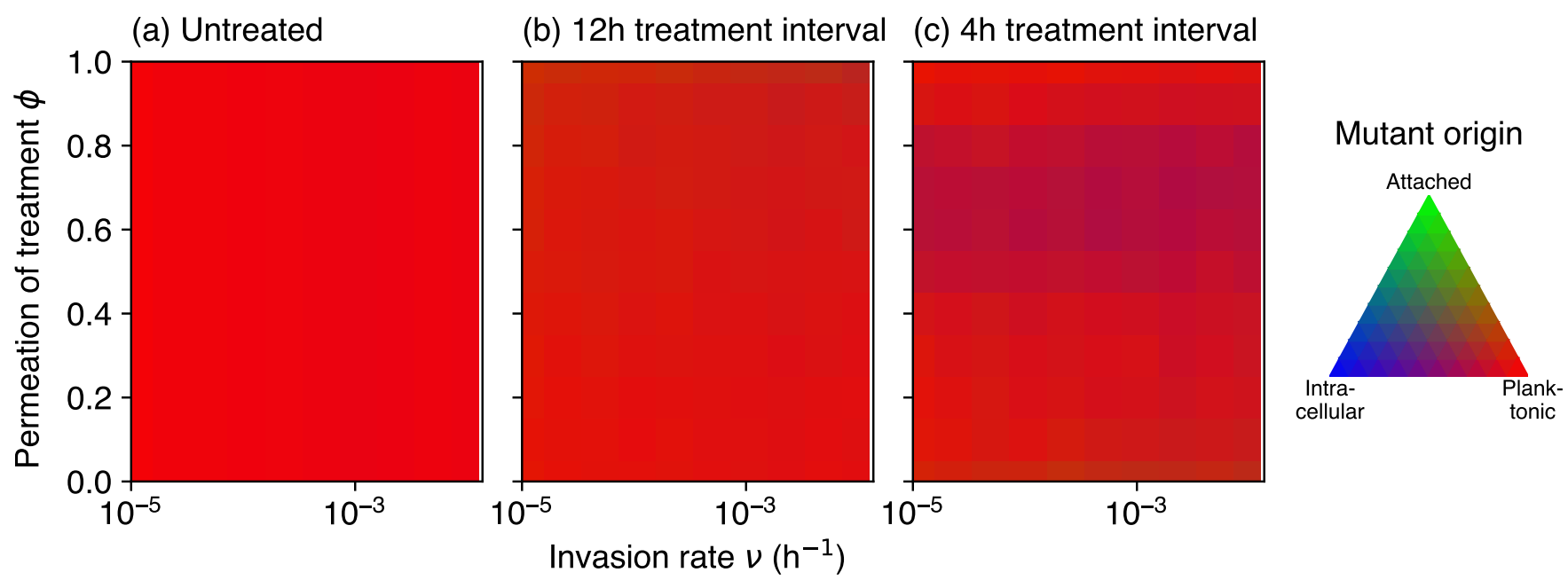


Figure S11: **Origin of non-extinct mutants for** *K_p_* = 10^7^**.** Other parameters as in Fig. 5.


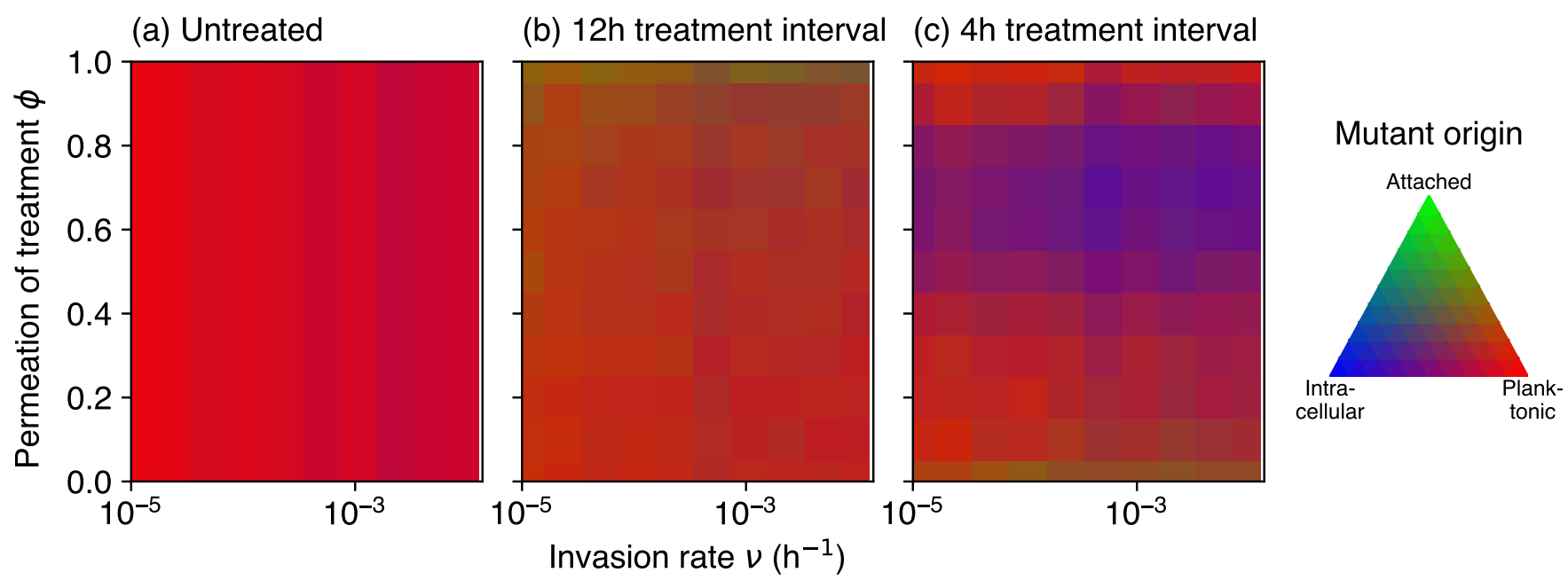


Figure S12: **Origin of non-extinct mutants for** *K_p_* = 3 *·* 10^6^**.** Other parameters as in Fig. 5.


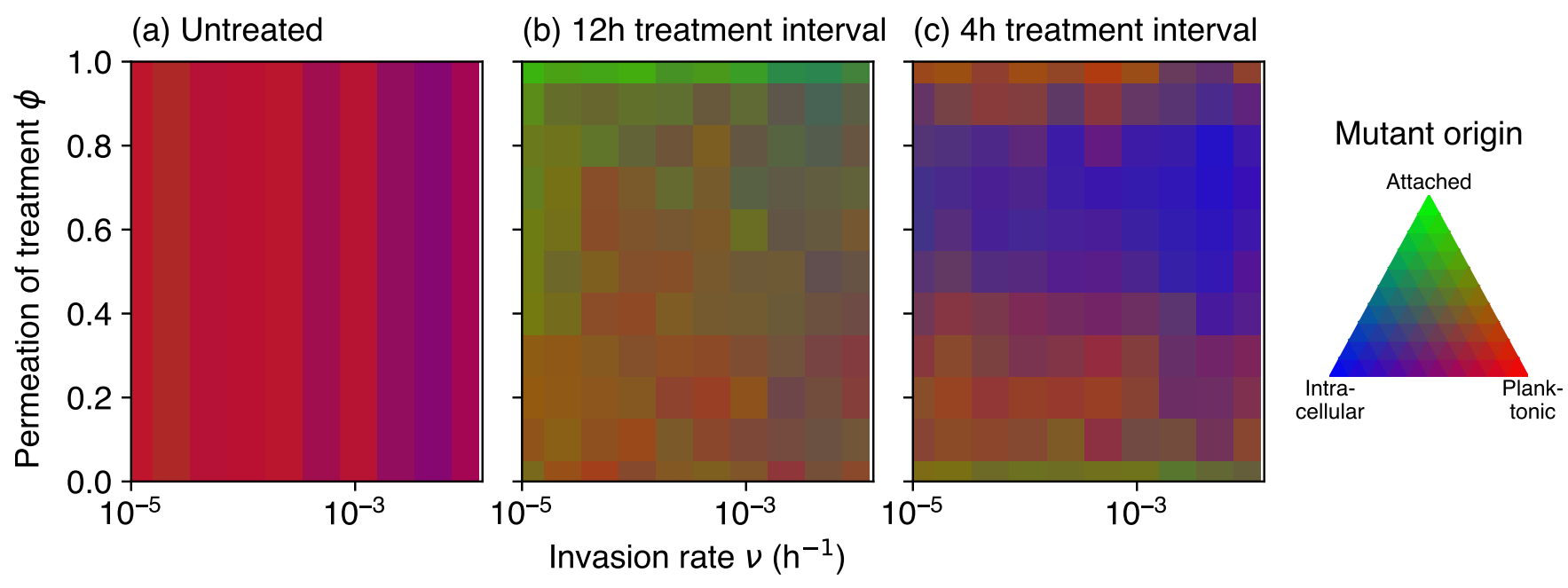


Figure S13: **Origin of non-extinct mutants f****or** *λ_d_* = 0 **(no detachment).** Other parameters as in Fig. 5, except that the number of simulated replicates is 200.

# References

- 1. Horsley H, Dharmasena D, Malone-Lee J, et al. A urine-dependent human urothelial organoid offers a potential alternative to rodent models of infection. Sci Rep. 2018;8(1).
  2. Jafari NV, Rohn JL. An immunoresponsive three-dimensional urine-tolerant human urothelial model to study urinary tract infection. Front Cell Infect Microbiol. 2023;13.
  3. Lin AE, Beasley FC, Olson J, et al. Role of hypoxia inducible factor-1*α* (HIF-1*α*) in innate defense against uropathogenic *Escherichia coli* infection. PLoS Pathog. 2015;11(4):e1004818.
  4. Islam K Md Jahirul Bagale, John PP, et al. Glycosuria alters uropathogenic *Escherichia coli*

global gene expression and virulence. mSphere. 2022;7(3):e00004–22.

- 1. Andersen-Civil S Audrey Inge Schytz Ahmed, Guerra PR, et al. The impact of inactivation of

the purine biosynthesis genes, purN and purT, on growth and virulence in uropathogenic *E. coli*. Mol Biol Rep. 2018;45(6):2707–2716.

- 1. Beebout CJ, Robertson GL, Reinfeld BI, et al. Uropathogenic *Escherichia coli* subverts mitochondrial metabolism to enable intracellular bacterial pathogenesis in urinary tract infection. Nat Microbiol. 2022;7(9):1348–1360.
  2. Kurimura Y, Nishitani C, Ariki S, et al. Surfactant protein D inhibits adherence of uropathogenic *Escherichia coli* to the bladder epithelial cells and the bacterium-induced cytotoxicity: a possible function in urinary tract. J Biol Chem. 2012;287(47):39578–39588.
  3. Seiler C, van Velzen E, Neu TR, et al. Grazing resistance of bacterial biofilms: a matter of predators’ feeding trait. FEMS Microbiol Ecol. 2017;93(9):fix112.
  4. Hirakawa H, Shimokawa M, Noguchi K, et al. The PapB/FocB family protein TosR acts as a positive regulator of flagellar expression and is required for optimal virulence of uropathogenic *Escherichia coli*. Front Microbiol. 2023;14.
  5. Abell-King C, Pokhrel A, Rice SA, et al. Bacterial multispecies invasion of human epithelial bladder cells. bioRxiv. 2023.
  6. Komp Lindgren P, Karlsson Å, Hughes D. Mutation rate and evolution of fluoroquinolone resistance in *Escherichia coli* isolates from patients with urinary tract infections. Antimicrob Agents Chemother. 2003;47(10):3222–3232.
  7. Sharma K, Thacker VV, Dhar N, et al. Early invasion of the bladder wall by solitary bacteria protects UPEC from antibiotics and neutrophil swarms in an organoid model. Cell Rep. 2021;36(3):109351.
  8. Sharma K, Dhar N, Thacker VV, et al. Dynamic persistence of UPEC intracellular bacterial communities in a human bladder-chip model of urinary tract infection. eLife. 2021;10:e66481.
